# CONNECTIVITY ISSUES WHEN CONDUCTING A VIRTUAL CLINICAL TRIAL WITH FAMILY CAREGIVERS IN RURAL OR UNDERSERVED AREAS

**DOI:** 10.1093/geroni/igae098.2114

**Published:** 2024-12-31

**Authors:** Diane Holland, Catherine Vanderboom, Jay Mandrekar, Allison Gustavson, Brystana Kaufman, Joan Griffin, Ellen Wild, Ann Marie Dose

**Affiliations:** Mayo Clinic, Rochester, Minnesota, United States; Mayo Clinic, Rochester, Minnesota, United States; Mayo Clinic, Rochester, Minnesota, United States; Minneapolis Veterans Affairs Health Care System, Minneapolis, Minnesota, United States; Duke University School of Medicine, Durham, North Carolina, United States; Mayo Clinic, Rochester, Minnesota, United States; Mayo Clinic, Rochester, Minnesota, United States; Mayo Clinic, Rochester, Minnesota, United States

## Abstract

In the research literature and amongst grant reviewers, there are persistent questions about whether internet connectivity challenges, especially in rural populations, will limit the potential for virtual or decentralized clinical trials to improve participation of underrepresented groups. In clinical practice, e-health and telehealth advancements have been touted as potential models of care to address health disparities associated with access to care, but without reliable internet connectivity, these disparities may perpetuate or even widen. In this presentation Dr. Holland examines the prevalence of connectivity problems during the Technology-Enhanced Transitional Palliative Care (TPC) study, a virtual clinical trial across three Midwestern states, and provides guidance on the challenges faced. Data are from structured notes from nurse interventionists who documented any connectivity issues in the study’s electronic health record. Of the 1003 visits reviewed, only 11% of visits (115/1003) contained a documented problem with internet connectivity. None of the participants withdrew from the trial due to problems with their internet connections. The findings support the effective use of virtual visits in research involving participants living in rural locations. Virtual randomized controlled trials provide a strategy that enables participation for rural individuals who may not otherwise have access to clinical trials conducted in-person in urban settings. Utilizing internet access to connect with and support people who live in rural areas is critically needed to advance clinical research.

